# Usefulness of the autism spectrum quotient (AQ) in screening for autism spectrum disorder and social communication disorder

**DOI:** 10.1186/s12888-023-05362-y

**Published:** 2023-11-13

**Authors:** Kiyohiro Yoshinaga, Jun Egawa, Yuichiro Watanabe, Hiroyuki Kasahara, Atsunori Sugimoto, Toshiyuki Someya

**Affiliations:** 1 Department of Psychiatry, Niigata University Graduate School of Medical and Dental Sciences, 757 Asahimachidori-ichibancho, Chuo-ku, Niigata, 951-8510 Japan; 2 Department of Psychiatry, Niigata Psychiatric Center, 2-4-1 Kotobuki, Nagaoka, 940-0015 Japan; 3 Department of Community Psychiatric Medicine, Niigata University Graduate School of Medical and Dental Sciences, 757 Asahimachidori-ichibancho, Chuo-ku, Niigata, 951-8510 Japan; 4 Department of Psychiatry, Uonuma Kikan Hospital, 4132 Urasa, Minimiuonuma, Niigata 949-7302 Japan

**Keywords:** Autism spectrum quotient, Autism spectrum disorder, Social (pragmatic) communication disorder, Receiver operating characteristic analysis

## Abstract

**Background:**

In the Diagnostic and Statistical Manual and Mental Disorders, Fifth Edition (DSM-5), autism spectrum disorder (ASD) and social (pragmatic) communication disorder (SCD) were described as a new category of psychiatry nosography. SCD involves impairments in social communication and social interaction but not restricted, repetitive patterns of behavior, interests, or activities. The autism spectrum quotient (AQ) was developed to screen for autism tendencies in adults with normal intelligence. However, AQ cutoff scores for screening ASD and SCD in the DSM-5 have not been established. This study examined whether the Japanese version of the AQ (AQ-J) total scores could discriminate between an ASD group, an SCD group, and a neurotypical (NT) group.

**Methods:**

Participants were 127 ASD patients, 52 SCD patients, and 49 NT individuals. Receiver operating characteristic (ROC) analyses were used to examine AQ-J total score cutoff values to distinguish between ASD and NT groups, SCD and NT groups, and ASD and SCD groups.

**Results:**

In the ROC analysis for the ASD and NT groups, the area under the curve (AUC) was 0.96, and the optimum cutoff value was 23 points (sensitivity 92.9%, specificity 85.7%). The AUC for the SCD and NT groups was 0.89, and the optimum cutoff value was 22 points (sensitivity 84.6%, specificity 85.7%). The AUC for the ASD and SCD groups was 0.75; the optimum cutoff value was 32 points (sensitivity 67.7%, specificity 71.2%).

**Conclusion:**

Our findings suggest the usefulness of the AQ-J in screening for ASD and SCD.

**Supplementary Information:**

The online version contains supplementary material available at 10.1186/s12888-023-05362-y.

## Introduction

In the Diagnostic and Statistical Manual and Mental Disorders, Fifth Edition (DSM-5), which was revised in 2014, conditions previously diagnosed as autistic disorder, Asperger’s disorder, and unspecified pervasive developmental disorder were unified into autism spectrum disorder (ASD) [1]. Social (pragmatic) communication disorder (SCD) is a new disease concept included in the DSM-5, which involves impairments in social communication and social interaction but not restricted, repetitive patterns of behavior, interests, or activities (RRBs) [2]. SCD is included in the macro category of communication disorders, characterized by a primary difficulty with broadly conceived pragmatic abilities, including language disorders, speech sound disorders, and childhood-onset fluency disorders (stuttering), but not ASD [3]. Although the distinction between SCD and ASD is controversial, at least the DSM-5 indicates that ASD and SCD are independent diagnostic concepts, so it is worthwhile to distinguish between these groups and examine whether there are differences in prognosis and comorbidities.

The Autism Spectrum Quotient (AQ) created by Baron-Cohen et al. (2001) is a self-response screening tool for autism tendencies in adults with normal intelligence. Based on the autism spectrum hypothesis, this scale can be used not only for clinical screening to determine whether or not an individual fits ASD, the degree of the disorder, and whether a precise diagnosis should be made but also to measure individual differences in autistic tendencies in normal subjects, which is considered beneficial in both diagnosis and research. A systematic review focused on the AQ analyzed 73 papers, including 6,934 nonclinical participants and 1,963 clinical cases with matched autism spectrum condition (ASC) [4]. The results showed that the mean AQ score was 17.0 (confidence interval [CI]: 16.4 to 17.4) in the nonclinical group and 35.2 (CI: 34.5 to 35.9) in the ASC group [5]. However, few studies have investigated the AQ cutoff score for screening ASD in the DSM-5 [6], and no studies have attempted to apply the AQ to screen SCD. This study examined whether total scores in the Japanese version of the AQ (AQ-J) could discriminate between an ASD group, an SCD group, and a neurotypical (NT) group.

## Methods

### Participants

This study was conducted in accordance with the Declaration of Helsinki and approved by the Institutional Review Board of Niigata University (approval number: 2019-0054). All participants received an explanation of the research content and provided written informed consent to participate.

Participating patients were outpatients at the Niigata University Medical and Dental Hospital and diagnosed with ASD or SCD according to the DSM-5. Diagnoses were made by experienced psychiatrists based on all available information, including unstructured interviews with participants and their families, clinical observation, and examination of medical records, including AQ score. There were 127 participants in the ASD group (39 females, mean age 28.0 ± 9.7 years) and 52 participants in the SCD group (20 females, mean age 28.1 ± 9.1 years). The information on comorbidities was in the supplemental material (Supplementary Table 1). The NT group comprised 49 participants (26 females, mean age 30.3 ± 10.1 years) who were recruited from the general population through community advertisements in the local area. These NT participants had no academic or occupational problems and a history of mental illness by self-report. All participants were confirmed to be free of intellectual disability (intelligence quotient [IQ] > 70) by intelligence tests.

**Table 1:** Total and subscale scores for the Japanese version of the autism spectrum quotient in the ASD, SCD, and NT groups

	Group				P			
	ASD	SCD	NT		Overall	Post-hoc		
						ASD vs. SCD	ASD vs. NT	SCD vs. NT
Female/male	39/88	20/32	26/23		0.0224^a^	–	–	–
Age, years (SD) range	28.0 (9.69) 16–59	28.1 (9.06) 16–52	30.3 (10.11) 16–59		0.3400^b^	–	–	–
IQ (SD) range	94.8 (14.0)	97.2 (14.9)	105.9 (7.7)		< 0.001^b^	0.4945	< 0.001	0.0029
AQ total score range	33.80 (7.00) 15–57	27.65 (6.15) 13–38	14.51 (7.75) 1–34		< 0.001^b^	< 0.001	< 0.001	< 0.001
Social skills	7.71(2.19)	7.07 (2.23)	2.73 (2.41)		< 0.001^b^	0.201	< 0.001	< 0.001
Attention switching	7.48 (1.84)	6.11 (1.67)	3.59 (2.15)		< 0.001^b^	< 0.001	< 0.001	< 0.001
Attention to detail	4.83 (2.16)	3.48 (1.73)	3.61 (2.30)		< 0.001^b^	< 0.001	0.002	0.948
Communication	7.48 (2.13)	5.98 (2.37)	1.98 (2.19)		< 0.001^b^	< 0.001	< 0.001	< 0.001
Imagination	6.14 (2.12)	5.00 (2.03)	2.61 (1.86)		< 0.001^b^	0.002	< 0.001	< 0.001

Data expressed as mean (standard deviation). The level of significance was set at an overall p < 0.0056 based on Bonferroni correction for nine tests. We applied the Tukey correction to post-hoc tests to identify significant differences between groups

Abbreviations: ASD, autism spectrum disorder; IQ, intelligence quotient; NT, neurotypical; SCD, social communication disorder

^a^ Calculated using a χ^2^ test

^b^ Calculated using analysis of variance

### Measurements

The AQ comprises 50 items, with each item answered on a scale from 1 to 4; depending on each item, response options 1 and 2 may be counted as 1 point, or options 3 and 4 may be counted as 1 point. AQ-J total scores range from 0 to 50 points, with high scores indicating high autistic traits. Baron-Cohen et al. (2001) classified the 50 AQ items into five 10-item subscales: social skills, attention switching, attention to detail, communication, and imagination [4]. Wakabayashi et al. (2006) standardized the AQ-J and set the cutoff value as 33 points [7]. We calculated the AQ-J total score and scores for the five subscales for each participant.

Intelligence tests were performed for participants using the Wechsler Adult Intelligence Scale (WAIS) 3rd or 4th edition [8–10], the Wechsler Intelligence Scale for Children (WISC) 3rd or 4th edition [11–13], or the Japanese version of the National Adult Reading Test (JART) [14]. The JART is a standardized cognitive function test for estimating premorbid IQ in patients with cognitive impairment [15]. These tests confirmed that the IQ of all participants was > 70.

### Statistical analyses

First, we compared the sex ratio, age, IQ scores, and AQ-J total and subscale scores among the three groups (ASD, SCD, and NT) using χ^2^ tests and analysis of variance (ANOVA). The significance level was set at p < 0.0056 based on Bonferroni correction for nine tests. When significant differences were found among the three groups in the ANOVA, we performed post hoc tests to identify significant differences between groups with Tukey correction.

Second, receiver operating characteristic (ROC) analyses were performed for (1) the ASD and NT groups, (2) the SCD and NT groups, and (3) the ASD and SCD groups. A ROC curve was drawn based on the average of the total scores for each group, and the area under the curve (AUC) was measured to estimate the optimum cutoff value. The score at the point closest to the coordinate (0, 1) in the upper left corner of the ROC curve was defined as the optimal cutoff value [16, 17]. All statistical analyses were conducted using BellCurve for Excel software.

## Results

**Fig. 1:**
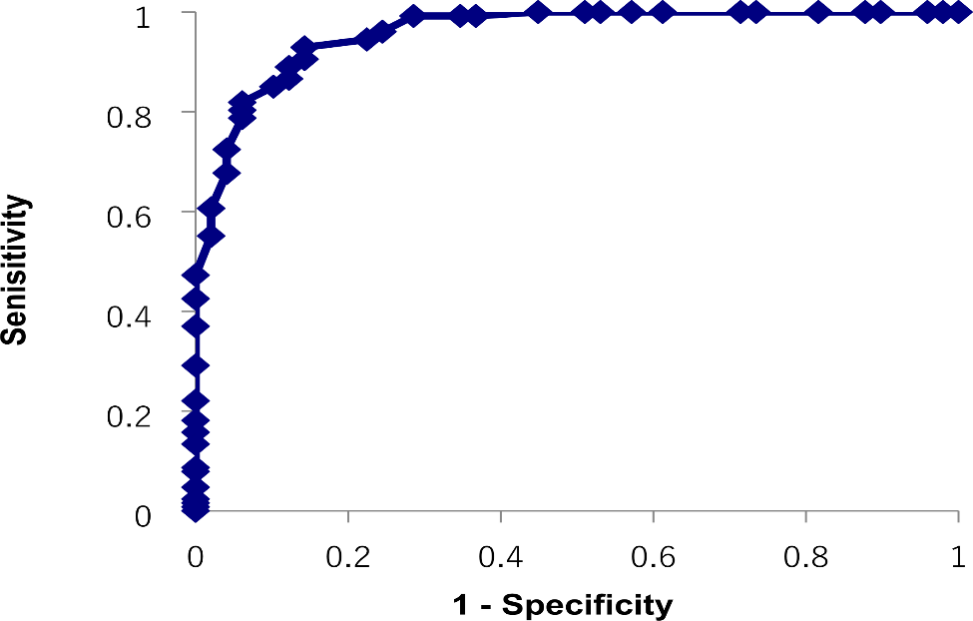
Receiver operating characteristic curve for the Japanese version of the autism spectrum quotient total score to distinguish between the autism spectrum disorder and neurotypical groups

**Fig. 2:**
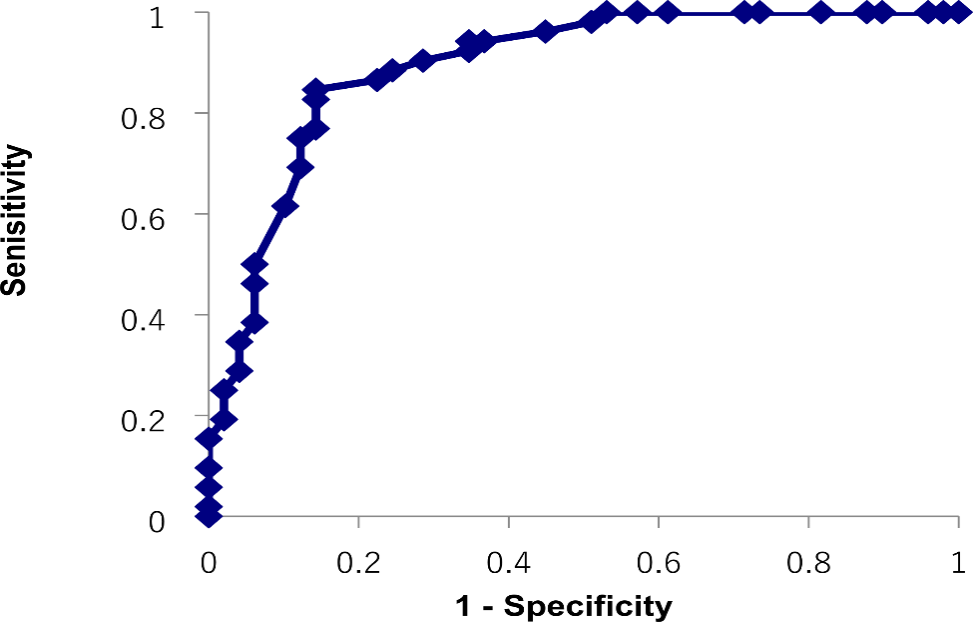
Receiver operating characteristic curve for the Japanese version of the autism spectrum quotient total score to distinguish between the social communication disorder and neurotypical groups

**Fig. 3:**
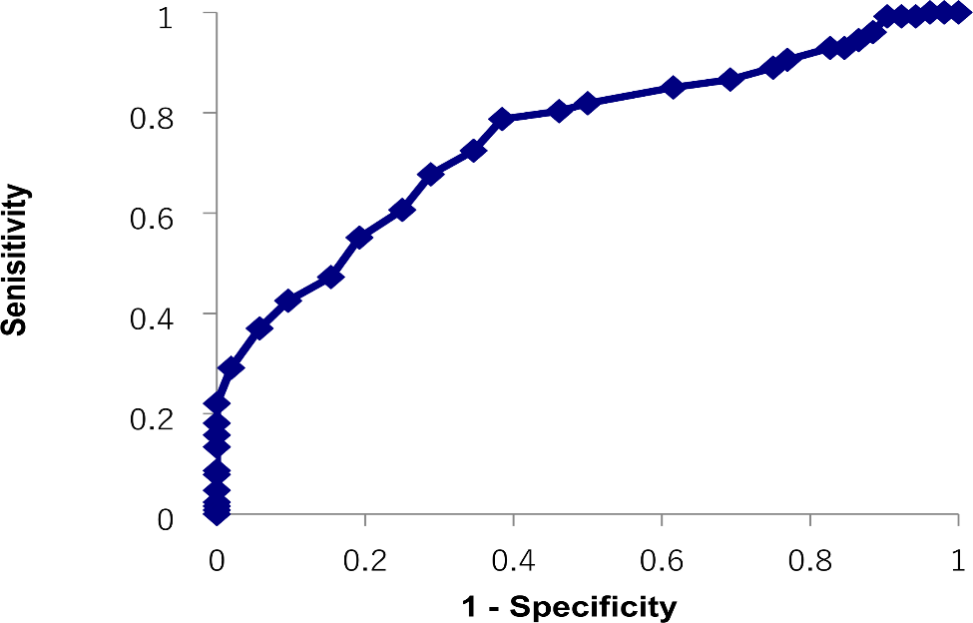
Receiver operating characteristic curve for the Japanese version of the autism spectrum quotient total score to distinguish between the autism spectrum disorder and social communication disorder groups

There were no significant differences in the sex ratio or average age among the three groups (Table 1). Significant differences were found among the three groups in IQ scores and the AQ-J total and subscale scores (p < 0.001 for all). Next, we performed post hoc tests. In the comparison between the ASD and SCD groups, significant differences were found in the AQ-J total and four subscales (attention switching, attention to detail, communication, and imagination) scores. In the comparison between the ASD and NT groups, significant differences were found in the IQ scores and AQ-J total and all five subscale scores. In the comparison between the SCD and NT groups, significant differences were found in IQ scores and AQ-J total and four subscales (social skills, attention switching, communication, and imagination) scores. In the ROC analysis for the ASD and NT groups, the AUC was 0.96, and the optimal cutoff value was 23 points (sensitivity 92.9%, specificity 85.7%, Positive Predictive Value (PPV) 94.4%, Negative Predictive Values (NPVs) 82.3%) (Fig. 1). The AUC for the SCD and NT groups was 0.89, and the optimum cutoff value was 22 points (sensitivity 84.6%, specificity 85.7%, PPV 86.3%, NPV 84.0%) (Fig. 2). The AUC for the ASD and SCD groups was 0.74, and the optimum cutoff value was 32 points (sensitivity 67.7%, specificity 71.1%, PPV 85.1%, NPV 52.6%) (Fig. 3). Similar analyses for men only were performed with similar results (Supplementary Table 2, Supplementary Figure 1).

## Discussion

In the differentiation between the ASD and NT groups, the AUC was 0.96, which indicated high accuracy (0.9–1.0) [18], and the optimum cutoff value was 23. Ko et al. (2018) also reported that the optimum cutoff value of the Korean version of AQ was 23, distinguishing between 20 patients with ASD without intellectual disability and 99 NT individuals in the Korean population [6]. However, a previous Japanese study reported that the cutoff value of the AQ-J was 33 points [7]. The case group in that study included patients with autistic disorder without intellectual disability and Asperger’s disorder using DSM-IV criteria, excluding unspecified pervasive developmental disorder which does not meet the full diagnostic criteria for autistic disorder or Asperger’s disorder, but some of the symptoms are present. The AQ-J total score (37.9 ± 5.31) for the case group in the previous study was higher than the score (33.80 ± 7.00) for the ASD group in our study. ASD characteristics are widely distributed throughout the population [19], but are often overlooked by medical, educational, and social service professionals. This creates barriers to accessing the services needed to help such individuals become independent [20]. Although a lower cutoff value increases the risk of false positives, we considered the AQ-J a useful tool in the primary care context to ensure that those needing help are not missed.

The SCD group did not differ significantly from the NT group in the attention to detail score related to RRBs, which is the main symptom of ASD; however, there was no significant difference from the ASD group only in the score for social skills (Table 1). The position of SCD in the diagnostic classification is still under debate, especially in differentiating SCD from ASD [3, 21]. The AUCs for the SCD and NT groups and the SCD and ASD groups were 0.89 and 0.75, respectively, representing relatively good values with moderate accuracy (0.7–0.9) [18]. These results suggested that SCD can be distinguished from both NT and ASD using the AQ-J total score. No studies on SCD screening have been reported, and it is worthwhile to demonstrate the usefulness of AQ, the world’s most commonly used autism screening tool, in this context.

Our study had some limitations that should be considered. First, patients were not assessed by standardized structured interviews such as the Autism Diagnostic Interview-Revised [22] or Autism Diagnostic Observation Schedule [23], although no structured interview tool is available for SCD. Second, the measurements for IQ used in this study included standard intelligence tests (WAIS or WISC) as well as the JART. However, even if the IQ estimate based on the JART was inaccurate, there was no occupational or academic maladaptation in the NT group, and the ASD and SCD groups were not diagnosed as having an intellectual disability by the psychiatrist interviews. Therefore, it is unlikely that the study participants included patients with intellectual disabilities. Third, significant differences in IQ were found among the groups (ASD vs. NT and SCD vs. NT), although we excluded individuals with an IQ < 70 from this study. Fourth, our study was the unequal sample sizes between SCD and ASD participants, reflecting the natural prevalence difference between these disorders. The smaller SCD sample may affect the robustness of our analyses. Fifth, to establish that the AQ can be used for screening, it is necessary to conduct an AQ in the general population and conduct a diagnostic interview study of at least those cases in which the AQ exceeds the cutoff, but this study included pre-diagnosed case controls. Prior studies exist on whether AQ can differentiate schizophrenia and ADHD from ASD, but these studies also used pre-diagnosed case samples, which is an issue for the future [24, 25]. Lastly, the study is a retrospective case-control design, and even though the clinicians who conducted the diagnostic evaluation based their diagnosis not only on the AQ score but also on clinical information obtained from interviews with the patients themselves, their families, and others around them, this is not a prospective cohort design blinded for the AQ score, which may be a confounding factor.

## Conclusion

The ROC analyses estimated the AQ-J tentative cutoff points of 23 for the ASD and NT groups, 22 for the SCD and NT groups, and 32 for the ASD and SCD groups. These findings suggest the usefulness of the AQ-J in screening for ASD and SCD.

### Electronic supplementary material

Below is the link to the electronic supplementary material.

Supplementary Material 1: **Table 1**. Comorbidities in ASD and SCD patients. **Table 2**. Total and subscale scores for the Japanese version of the autism spectrum quotient in the ASD, SCD, and NT groups (male samples only). **Figure 1**. Receiver operating characteristic curve for the Japanese version of the Autism Spectrum Quotient total score to distinguish between the autism spectrum disorder and neurotypical groups in males. The AUC was 0.95. **Figure 2**. Receiver operating characteristic curve for the Japanese version of the Autism Spectrum Quotient total score to distinguish between the social communication disorder and neurotypical groups in males. The AUC was 0.87. **Figure 3**. Receiver operating characteristic curve for the Japanese version of the Autism Spectrum Quotient total score to distinguish between the autism spectrum disorder and social communication disorder groups in males. The AUC was 0.73

Supplementary Material 2: Demographic and clinical data of typically developing individuals

## Data Availability

The datasets analyzed during the current study are not publicly available due to privacy and confidentiality concerns. However, anonymized data may be made available from the corresponding author upon reasonable request. Materials used in the study are available upon request.
