# Supplementary material for: Usefulness of the autism spectrum quotient (AQ) in screening for autism spectrum disorder and social communication disorder
Source: BMC Psychiatry. 2023 Nov 13;23:831. doi: 10.1186/s12888-023-05362-y (PMC10644653; doi:10.1186/s12888-023-05362-y)
Supplement: Supplementary file 1 — Supplementary Material 1: Table 1. Comorbidities in ASD and SCD patients. Table 2. Total and subscale scores for the Japanese version of the autism spectrum quotient in the ASD, SCD, and NT groups (male samples only). Figure 1. Receiver operating characteristic curve for the Japanese version of the Autism Spectrum Quotient total score to distinguish between the autism spectrum disorder and neurotypical groups in males. The AUC was 0.95. Figure 2. Receiver operating characteristic curve for the Japanese version of the Autism Spectrum Quotient total score to distinguish between the social communication disorder and neurotypical groups in males. The AUC was 0.87. Figure 3. Receiver operating characteristic curve for the Japanese version of the Autism Spectrum Quotient total score to distinguish between the autism spectrum disorder and social communication disorder groups in males. The AUC was 0.73 [file 12888_2023_5362_MOESM1_ESM.docx]

**Supplementary Table 1.** Comorbidities in ASD and SCD patients

|  | ASD | SCD | p-value |
| --- | --- | --- | --- |
| ADHD | 25 (19.7%) | 10 (19.2%) | > 0.05 |
| Trauma- and Stressor-Related Disorders | 6 (4.7%) | 4 (7.7%) | > 0.05 |
| Depressive Disorders | 5 (3.9%) | 8 (15.3%) | 0.018 |
| Anxiety Disorders | 4 (3.1%) | 0 | > 0.05 |
| Obsessive-Compulsive and Related Disorder | 3 (2.4%) | 0 | > 0.05 |
| Schizophrenia Spectrum and Other Psychotic Disorders | 2 (1.6%) | 0 | > 0.05 |
| Dissociative Disorders | 2 (1.6%) | 0 | > 0.05 |
| Specific Learning Disorder | 1 (0.8%) | 3 (5.8%) | > 0.05 |
| Somatic Symptom and Related Disorders | 1 (0.8%) | 1 (1.9%) | > 0.05 |
| Neurocognitive Disorders | 1 (0.8%) | 0 | > 0.05 |
| Childhood-Onset Fluency Disorder (Stuttering) | 1 (0.8%) | 0 | > 0.05 |
| Personality Disorders | 1 (0.8%) | 0 | > 0.05 |
| Substance-Related and Addictive Disorders | 1 (0.8%) | 1 (1.9%) | > 0.05 |
| Feeding and Eating Disorders | 1 (0.8%) | 0 | > 0.05 |

Figures in parentheses indicate percentages.

* The difference in the rate of comorbidity between the two groups was tested by the chi-square test or Fisher's exact probability test. 　 The significance level was set at 0.05/14 = 0.0036 using Bonferroni's correction for multiple comparisons.

**Supplementary Table 2.** Total and subscale scores for the Japanese version of the autism spectrum quotient in the ASD, SCD, and NT groups (male samples only)

|  | Group | | |  | P | | | |
| --- | --- | --- | --- | --- | --- | --- | --- | --- |
|  | ASD | SCD | NT |  | Overall | Post-hoc | | |
|  | N=87 | N=29 | N=23 |  |  | ASD vs. SCD | ASD vs. NT | SCD vs. NT |
| Age, years (SD)  range | 27.6 (9.44)  16-55 | 30.8 (10.31)  16-52 | 31.4 (10.08)  21-59 |  | 0.133^b^ | – | – | – |
| IQ (SD)  range | 94.2 (13.6) | 95.9 (16.7) | 106.8 (7.9) |  | <0.001^b^ | 0.4945 | <0.001 | 0.0029 |
| AQ total score  range | 34.00 (6.79)  19-47 | 29.3 (4.94)  20-38 | 15.3 (7.85)  5-32 |  | <0.001^b^ | 0.004 | <0.001 | <0.001 |
| Social skills | 7.75(2.06) | 7.79 (1.74) | 2.74 (2.50) |  | <0.001^b^ | 0.994 | <0.001 | <0.001 |
| Attention switching | 7.63 (1.77) | 6.34 (1.56) | 3.74 (2.14) |  | <0.001^b^ | 0.003 | <0.001 | <0.001 |
| Attention to detail | 4.89 (2.30) | 3.07 (1.49) | 3.22 (1.91) |  | <0.001^b^ | <0.001 | 0.003 | 0.965 |
| Communication | 7.44 (2.25) | 6.62 (2.09) | 2.57 (2.48) |  | <0.001^b^ | 0.214 | <0.001 | <0.001 |
| Imagination | 6.30 (2.09) | 5.52 (2.03) | 3.09 (1.83) |  | <0.001^b^ | 0.177 | <0.001 | <0.001 |
|  |  |  |  |  |  |  |  |  |

Data expressed as mean (standard deviation). The level of significance was set at an overall p < 0.0056 based on Bonferroni correction for nine tests. We applied the Tukey correction to post‐hoc tests to identify significant differences between groups.

Abbreviations: ASD, autism spectrum disorder; IQ, intelligence quotient; NT, neurotypical; SCD, social communication disorder.

^a^ Calculated using a χ^2^ test.

^b^ Calculated using analysis of variance.

Supplementary Figure 1

Supplementary Figure 2

Supplementary Figure 3

Figure legends

Supplementary Figure 1. Receiver operating characteristic curve for the Japanese version of the Autism Spectrum Quotient total score to distinguish between the autism spectrum disorder and neurotypical groups in males. The AUC was 0.95.

Figure 2. Receiver operating characteristic curve for the Japanese version of the Autism Spectrum Quotient total score to distinguish between the social communication disorder and neurotypical groups in males. The AUC was 0.87.

Figure 3. Receiver operating characteristic curve for the Japanese version of the Autism Spectrum Quotient total score to distinguish between the autism spectrum disorder and social communication disorder groups in males. The AUC was 0.73.
